# Genetic manipulation of a metabolic enzyme and a transcriptional regulator increasing succinate excretion from unicellular cyanobacterium

**DOI:** 10.3389/fmicb.2015.01064

**Published:** 2015-10-06

**Authors:** Takashi Osanai, Tomokazu Shirai, Hiroko Iijima, Yuka Nakaya, Mami Okamoto, Akihiko Kondo, Masami Y. Hirai

**Affiliations:** ^1^RIKEN Center for Sustainable Resource ScienceYokohama, Japan; ^2^Department of Agricultural Chemistry, School of Agriculture, Meiji UniversityKawasaki, Japan; ^3^Biomass Engineering Program, RIKENYokohama, Japan; ^4^Department of Chemical Science and Engineering, Graduate School of Engineering, Kobe UniversityKobe, Japan

**Keywords:** cyanobacteria, metabolism, metabolomics, sigma factor, succinate

## Abstract

Succinate is a building block compound that the U.S. Department of Energy (DOE) has declared as important in biorefineries, and it is widely used as a commodity chemical. Here, we identified the two genes increasing succinate production of the unicellular cyanobacterium *Synechocystis* sp. PCC 6803. Succinate was excreted under dark, anaerobic conditions, and its production level increased by knocking out *ackA*, which encodes an acetate kinase, and by overexpressing *sigE*, which encodes an RNA polymerase sigma factor. Glycogen catabolism and organic acid biosynthesis were enhanced in the mutant lacking *ackA* and overexpressing *sigE*, leading to an increase in succinate production reaching five times of the wild-type levels. Our genetic and metabolomic analyses thus demonstrated the effect of genetic manipulation of a metabolic enzyme and a transcriptional regulator on succinate excretion from this cyanobacterium with the data based on metabolomic technique.

## Introduction

In 2004, the U.S. Department of Energy (DOE) selected the top 12 building block chemicals from a list of more than 300 candidates that were produced from biomass (Werpy and Petersen, 2004). Among these, four-carbon dicarboxylic acids, including succinate, were included. Succinate can be used as a precursor to numerous chemicals such as a biodegradable plastic like polybutylene succinate, fibers, and pigments (Zeikus et al., 1999; Hong and Lee, 2002; Werpy and Petersen, 2004). Succinate is currently derived from petroleum, but it could also be produced using bacteria (McKinlay et al., 2007).

Production of succinate by recombinant heterotrophic bacteria such as *Escherichia coli, Corynebacterium glutamicum, Anaerobiospirillum succiniciproducens, Actinobacillus succinogenes*, and *Mannheimia succiniciproducens* has been intensively studied (Samuelov et al., 1991; Guettler et al., 1999; Chatterjee et al., 2001; Hong and Lee, 2001; Hong et al., 2004; Lee et al., 2006). Succinate is an intermediate in the tricarboxylic acid (TCA) cycle and is excreted by succinate-producing cells during anaerobic fermentation (McKinlay et al., 2007). Succinate is produced from phosphoenolpyruvate via the reductive branch of the TCA cycle, in which phosphoenolpyruvate is converted to oxaloacetate by phosphoenolpyruvate carboxylase (PEPC) or phosphoenolpyruvate carboxykinase (PEPCK) under anaerobic conditions (McKinlay et al., 2007). Malate is produced from oxaloacetate when catalyzed by malate dehydrogenase, and fumarate is produced from malate when catalyzed by fumarase. This is followed by the production of succinate when catalyzed by succinate dehydrogenase (SDH; McKinlay et al., 2007). The overexpression of a gene encoding PEPC in *E. coli* increases succinate production 3.8-fold (Millard et al., 1996). The introduction of PEPC, PEPCK, or malic enzyme (catalyzing a reaction from pyruvate to malate), also enhances succinate production in *E. coli* (Hong and Lee, 2001; Kim et al., 2004; Lin et al., 2005; Zhang et al., 2009). The deletion of *ldhA* (encoding L-lactate dehydrogenase), *adhE* (encoding alcohol dehydrogenase), and *ack-pta* (encoding acetate kinase and phosphotransacetylase, respectively) prevented the production of L-lactate, ethanol, and acetate, that are by-products during anaerobic fermentation, also increases the production of succinate in *E. coli* (Sánchez et al., 2005a,b; Jantama et al., 2007). In addition, the activation of the glyoxylate pathway by the deletion of iclR, which encodes the transcriptional repressor of the genes related to glyoxylate pathway, increased the succinate productivity in *E. coli* (Sánchez et al., 2005a,b). Thus, inhibition of by-product formation combined with additional genetic engineering can up-regulate succinate productivity.

Cyanobacteria are a group of bacteria that fix carbon dioxide via oxygenic photosynthesis. The potential applications of cyanobacteria in providing renewable energy and resources may reduce the environmental burden. Genome information for cyanobacteria is available (Kanesaki et al., 2012), and genetic engineering is easily performed by homologous recombination in several cyanobacterial strains, including the non-nitrogen fixing cyanobacterium *Synechocystis* sp. PCC 6803 (hereafter *Synechocystis* 6803; Ikeuchi and Tabata, 2001). The genome of *Synechocystis* 6803 was the first sequenced among the cyanobacteria (Kaneko et al., 1996), and it has been used extensively in basic and applied sciences.

**Figure 1:**
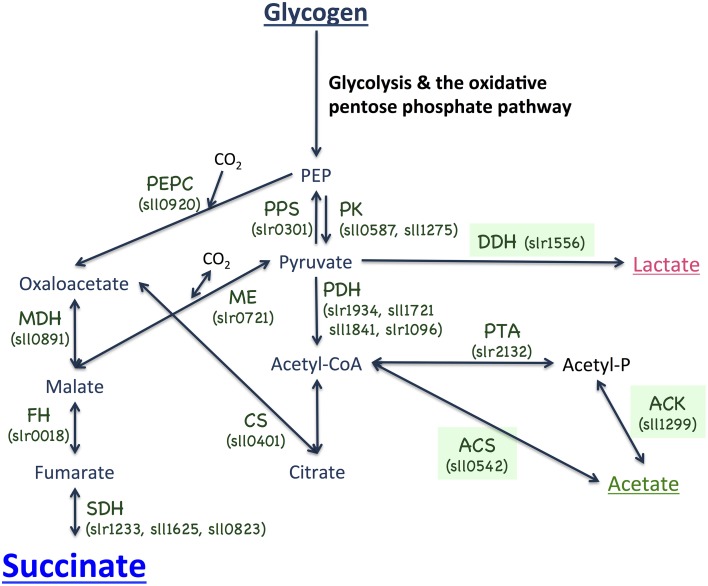
**Metabolic map surrounding succinate biosynthesis in the cyanobacterium ***Synechocystis*** sp. PCC 6803**. PEP, phosphoenolpyruvate; PEPC, phosphoenolpyruvate carboxylase; PPS, phosphoenolpyruvate synthase; PK, pyruvate kinase; MDH, malate dehydrogenase; ME, malic enzyme; FH, fumarate hydratase; SDH, succinate dehydrogenase; PDH, pyruvate dehydrogenase; DDH, D-lactate dehydrogenase, CS, citrate synthase; PTA, phosphotransacetylase; ACK, acetate kinase; and ACS, acetyl-CoA synthetase.

There are few reports of succinate production using cyanobacteria. McNeely et al. revealed that five fermentation products, lactate, acetate, succinate, alanine, and hydrogen, were produced under dark, anaerobic conditions by the marine cyanobacterium *Synechococcus* sp. PCC 7002 (hereafter *Synechococcus* 7002; McNeely et al., 2010). A knockout of *ldhA* increased acetate and hydrogen levels, and diminished lactate production (McNeely et al., 2010). Succinate was excreted from the *ldhA* knockout cells, but it was not detected from the wild-type cells of *Synechococcus* 7002 (McNeely et al., 2010). The filamentous, non-diazotrophic cyanobacteria *Arthrospira maxima* CS-328 cells produced lactate, acetate, ethanol, formate, and hydrogen under dark, anaerobic conditions, but succinate excretion was not detected (Carrieri et al., 2010, 2011). For *Synechocystis* 6803, hydrogen is generated under both light and dark, anaerobic conditions (Osanai et al., 2013). Organic acids, including D-lactates, were highly produced by genetically engineered *Synechocystis* 6803 cells, and succinate was also generated but its levels were only 3% of total carbon excreted, suggesting that genetic and metabolic engineering are necessary to increase succinate production (Angermayr et al., 2014; Hollinshead et al., 2014; McNeely et al., 2014). The genes encoding enzymes for organic acid production exist in the *Synechocystis* 6803 genome (Figure 1).

We reveal here that succinate was excreted from *Synechocystis* 6803 cells under dark, anaerobic conditions and the succinate levels were enhanced by reducing acetate biosynthesis and overexpressing *sigE* encoding a sigma factor. These results demonstrated the genetic manipulation of two types of genes increasing the succinate excretion from this cyanobacterium.

## Materials and methods

### Bacterial strains and culture conditions

The glucose-tolerant strain of *Synechocystis* sp. PCC 6803, isolated by Williams (Williams, 1988), was grown in modified BG-11 medium, consisting of BG-11_0_ liquid medium (Rippka, 1988) supplemented with 5 mM NH_4_Cl (buffered with 20 mM HEPES–KOH, pH 7.8). The GT-I strain, among GT substrains, was used in the current study (Kanesaki et al., 2012). Liquid cultures were bubbled with 1% (v/v) CO_2_ in air and incubated at 30°C under continuous white light (~50–70 μmol photons m^−2^ s^−1^). For the mutant strains, 10, 0.3, and 10 μg/mL of kanamycin, gentamycin and chloramphenicol, respectively, were added for preculturing. Modified BG-11 medium (containing 10 mM NH_4_Cl in liquid medium) was solidified with agar (1.5% w/v) for plate cultures, and similarly incubated in air at 30°C under continuous white light (~50–70 μmol photons m^−2^ s^−1^). Cell densities were measured at *A*_730_ using a Hitachi U-3310 spectrophotometer (Hitachi High-Tech., Tokyo, Japan).

For succinate production, cells grown in 70 mL modified BG-11 medium (started from *A*_730_ = 0.4) for 3 days were concentrated into 10 mL HEPES buffer (20 mM HEPES–KOH, pH 7.8) or modified BG-11 medium to *A*_730_ = 20 in a GC vial. The vial was sealed using butyl rubber, and N_2_ gas was introduced using syringes for 1 h to produce anaerobic conditions. After removing the syringes, the vial was wrapped with aluminum foil and shaken at 30°C. Cell cultures were then centrifuged at 5800 × *g* for 2 min, the supernatant was filtrated, and 1 mL supernatant was freeze-dried for 1 day. The dried sample was used for high-performance liquid chromatography analysis.

### Plasmid construction of knock-in vectors pTCP1556, pTCP0542, and pTCP1299

The kanamycin resistance cassette of the pTKP2031V vector (Osanai et al., 2011) was removed by digestion with *Xho*I and *Aat*II (Takara Bio, Shiga, Japan). The chloramphenicol resistance cassette from pKRP10 (Reece and Phillips, 1995) was amplified by PCR with KOD polymerase (Toyobo, Osaka, Japan) and the specific primers in Table S2, digested with *Xho*I and *Aat*II, and inserted into the *Xho*I-*Aat*II sites of pTKP2031V. The resultant plasmid was named pTCP2031. Regions of *ddh* (slr1556), from −297 to +800 bp, *acs* (sll0542), from +921 to +1962 bp, and *ackA* (sll1299), from +270 to +1238 bp, based on the translation initiation codons, were amplified by PCR with KOD plus neo polymerase (Toyobo) and the specific primers in Table S2. The fragments amplified by PCR were digested with *Sph*I and *Eco*RV (Takara Bio) and inserted into the *Sph*I-*Sma*I sites of the pUC119 vector (Takara Bio). The resultant plasmid was digested with *Hinc*II (for *ddh* and *ackA*) or *Apa*I (for *acs*), and the region including the chloramphenicol resistance cassette, *psbAII* promoter, and *Nde*I-*Hpa*I cloning sites of pTCP2031 was amplified with KOD plus neo polymerase and the specific primers 5′-TTTGCTTCATCGCTCGAG-3′ and 5′-ATCCAATGTGAGGTTAAC-3′, and integrated into the *Hinc*II or *Apa*I site of the plasmid. The resultant plasmids were named pTCP1556, pTCP0542, and pTCP1299 for knockouts of *ddh, acs*, and *ackA*, respectively. The *sigE* ORF was obtained by digestion with *Nde*I and *Hpa*I from pTGP0945-*sigE* plasmid (Osanai et al., 2014a) and cloned into the *Nde*I-*Hpa*I sites of pTCP1556, pTCP0542, and pTCP1299. The plasmids were integrated into the GT-I strain by natural transformation as described previously (Osanai et al., 2011). Knockouts and the insertion of the *sigE* ORF were confirmed by PCR using GoTaq (Promega, Fitchburg, WI, USA) with the primers in Table S2.

### Immunoblotting

Cells were collected by centrifugation (5800 × *g* for 2 min), and the supernatant was removed and cells were frozen by liquid nitrogen. Then, cells were dissolved in PBS-T and disrupted by sonication as described previously (Osanai et al., 2014a). Immunoblotting was performed as described previously (Osanai et al., 2014a). Antisera against SigE were generated previously (Osanai et al., 2009).

### Glycogen measurement

Glycogen levels were measured at the Biotechnology Center of Akita Prefectural University (Akita, Japan), as described in Osanai et al. (2014a).

### LC-MS/MS analysis

Equal amounts of cells (10 mL cell culture with *A*_730_ = 1.0) were harvested by rapid filtration, and metabolites were extracted using a previously described method (Osanai et al., 2014b). Briefly, the cells were filtrated, and then the intermediate metabolites were quenched and extracted in 1.2 mL of solvent mixture (CHCl_3_:CH_3_OH:H_2_O, 2.5:2.5:1, v/v/v) containing 10 μg/L D-(+)-camphor-10-sulfonic acid as an internal standard. After centrifugation at 15,000 × *g* at 4°C for 5 min, 400 μL of the upper phase was transferred to a new tube and vacuum-dried.

### GC-MS analysis

Equal amounts of cells (10 mL cell culture with *A*_730_ = 1.0) were harvested by rapid filtration as mentioned above. GC-MS was carried out using a GCMS-QP2010 Ultra, and the detailed protocol is described in Osanai et al. (2015).

### Measurement of organic acids by high-performance liquid chromatography (HPLC)

Freeze-dried supernatants were resolved in 100 μL of filtered 3 mM perchloric acid. The resolved samples were analyzed by HPLC using a LC-2000Plus Systems (JASCO, Tokyo, Japan) with a photodiode array detector and two RSpak KC-811 columns (Showa Denko, Tokyo, Japan). Organic acids were quantified with 0.2 mM bromothymol blue in 15 mM sodium phosphate buffer; peaks were detected at 445 nm. The column temperature was 60°C, and the flow rates of 3 mM perchloric acid and 0.2 mM bromothymol blue solutions were 1.0 and 1.5 mL/min, respectively.

## Results

### *ackA* knockout and *sigE* overexpression enhanced succinate production

**Figure 2:**
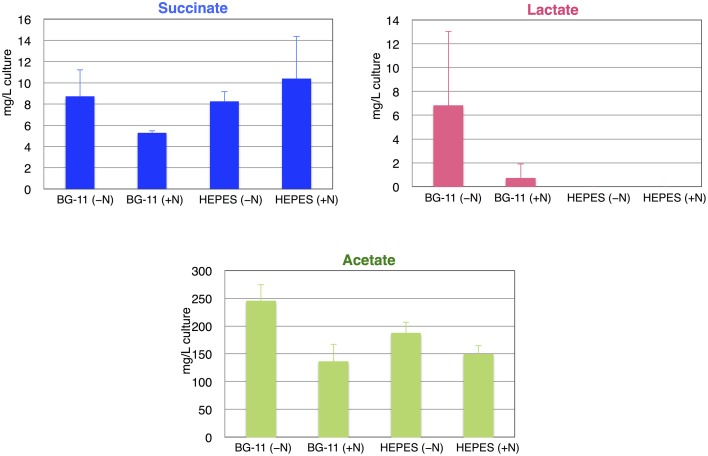
**Levels of succinate, lactate, and acetate from the wild-type cyanobacterium ***Synechocystis*** 6803 using different media or buffers during anaerobic conditions**. Organic acids excreted from 3 days under dark, anaerobic cultivation were quantified by HPLC. Data represent means ± SD from three independent experiments. +N designates 5 mM NH_4_Cl was added to the BG-11 medium or 20 mM HEPES–KOH (pH 7.8) buffer.

The identities of the excreted organic acids from the wild-type *Synechocystis* 6803 (GT) during anaerobic conditions were determined first. After cultivation for 3 days under light, aerobic conditions (1% CO_2_ in the air), cells were concentrated into 10 mL BG-11_0_ medium or HEPES buffer with or without nitrogen sources (5 mM NH_4_Cl) in a GC-vial, subjected to anaerobic conditions by introducing N_2_ gas, and incubated for 3 days under dark conditions with shaking at 30°C. Organic acids excreted into the medium or buffer were analyzed by HPLC. Succinate, lactate and acetate were detected, and the succinate levels were highest in HEPES buffer with nitrogen source among the four conditions tested (Figure 2). Lactates were not detected in HEPES buffer (Figure 2). Acetate levels were higher in BG-11 or HEPES buffer without nitrogen sources than those in BG-11 or HEPES buffer with nitrogen sources (Figure 2). To reduce the cost of succinate production, subsequent experiments were performed using HEPES buffer without nitrogen sources.

**Figure 3:**
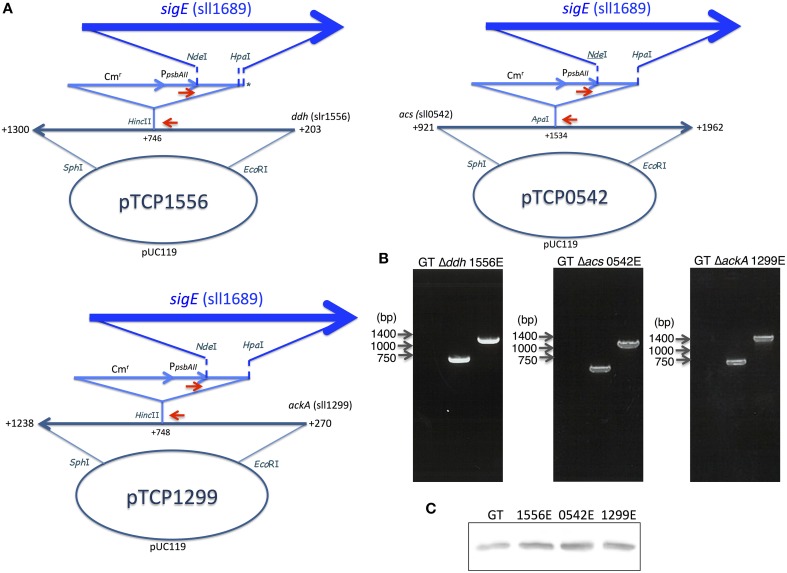
**(A)** Vector maps for three vectors disrupting *ddh, acs*, and *ackA* with or without *sigE* overexpression. Red arrows indicate the primer sets to confirm the insertion of the vectors in the non-nitrogen fixing cyanobacterium *Synechocystis* sp. PCC 6803 genome. Asterisk (^*^) in pTCP1556 indicates that the *sigE* ORF was inserted into the region from *Nde*I to 55-bp downstream of the *Hpa*I site because of the star activity of *Hpa*I. **(B)** The right bottom panels show DNA fragments amplified by PCR with the primers in agarose gel electrophoresis as visualized by ethidium bromide. **(C)** Protein levels of SigE in GT, 1556E, 0542E, and 1299E strains. Immunoblotting was performed with 12 μg of total protein from cells grown under dark, anaerobic conditions for 3 days.

To increase succinate production, we applied two strategies, decreasing lactate and acetate by knocking out each of the three genes (*ddh, acs*, or *ackA*; Figure 1) and promoting the sugar catabolic pathway by overexpressing *sigE*, encoding an RNA polymerase sigma factor, which activates the expression of sugar catabolic enzymes (Osanai et al., 2011). Knock-in vectors, which integrate the region containing the chloramphenicol resistance cassette, the *psbAII* promoter from the D1 protein of Photosystem II, and *Nde*I-*Hpa*I cloning sites, were constructed to generate the knockout mutants of *ddh, acs*, and *ackA* (Figure 3A). The *sigE* open reading frame (ORF) was cloned into the *Nde*I-*Hpa*I sites to generate the *sigE* overexpression strain combined with the *ddh, acs*, or *ackA* knockout, and the resultant strains were designated as 1556E, 0542E, and 1299E, respectively (Figure 3A). The insertion of these DNA fragments was confirmed by PCR (Figure 3B). Immunoblotting confirmed that SigE proteins in the three *sigE*-overexpressing strains were higher than in GT after 3 days of cultivation under dark, anaerobic conditions (Figure 3C).

**Figure 4:**
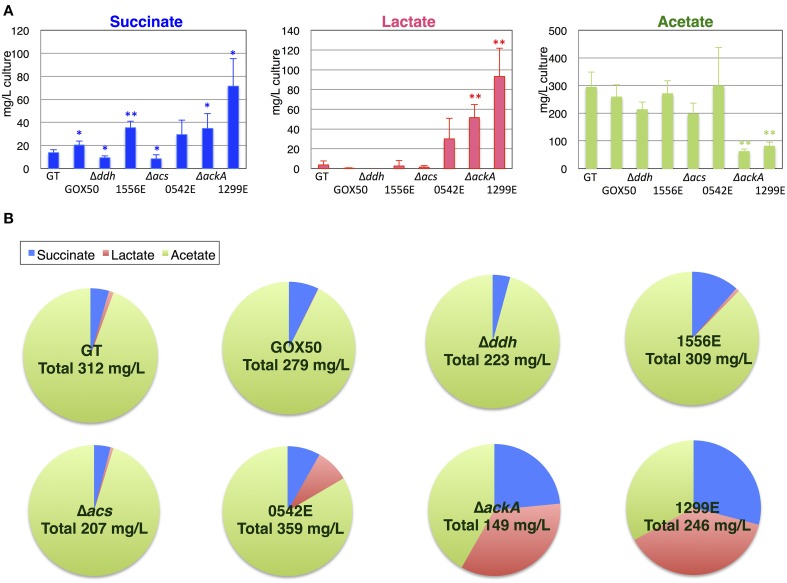
**Production of organic acids from the cyanobacterium ***Synechocystis*** 6803 strain overexpressing ***sigE*** and lacking ***ddh*** (slr1556), ***acs*** (sll0542), or ***ackA*** (sll1299)**. **(A)** Levels of organic acids excreted during 3 days of dark, anaerobic cultivation were quantified by HPLC. Δ*ddh*, Δ*acs*, and Δ*ddh* indicate the knockout of mutants of each gene. 1556E, 0542E, and 1299E represent the strains overexpressing *sigE* and lacking *ddh, acs*, or *ackA*, respectively. GOX50 designates the *sigE*-overexpressing strain. Data represent means ± SD from three or four independent experiments. Asterisks indicate statistically significant differences between GT and the mutant strains (Student's *t*-test; ^*^P < 0.05, ^**^P < 0.005). **(B)** The pie chart shows the ratio of succinate, lactate, and acetate excreted from the cells under anaerobic conditions. Total organic acid acids are sum of succinate, lactate, and acetate amounts excreted from the cells. Ratio is calculated by dividing the amount of each organic acid by the amounts of total organic acids.

**Figure 5:**
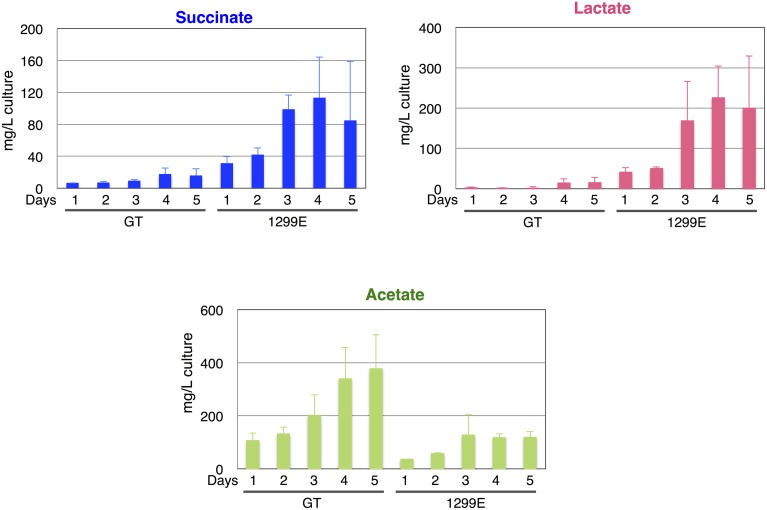
**Time-course analysis of levels of succinate, lactate, and acetate from the wild-type cyanobacterium ***Synechocystis*** 6803 and the 1299E strain, which overexpresses ***sigE*** and lacks ***ackA*****. Organic acids excreted from 1 to 5 days under dark, anaerobic cultivation were quantified by HPLC. Data represent means ± SD from three independent experiments.

Although the knockouts of *ddh* and *acs* did not increase the succinate levels, the *ackA* knockout increased the succinate level to 34.8 mg/L compared with the 13.9 mg/L produced by the parental wild-type strain under the same conditions. *sigE* overexpression (GOX50) alone increased the succinate level to 20.3 mg/L, and an additional knockout of *ddh, acs*, or *ackA* enhanced the levels to ~35.6, 29.4, or 71.5 mg/L, respectively (Figure 4A). The wild-type cells produced less than 10 mg/L lactate, while the lactate levels increased in the *ackA* knockout to 51.7 mg/L, and *sigE* overexpression with an *acs* or *ackA* knockout enhanced the levels to 30.0 or 93.5 mg/L, respectively (Figure 4A). Acetate levels were decreased to 62.0 mg/L by the *ackA* knockout, compared with 294.3 mg/L acetate produced by wild-type cells, and the ratio of succinate and lactate to acetate increased in the *ackA* knockout mutant (Figures 4A,B). The strain lacking *ackA* and overexpressing *sigE* (1299E) had the highest succinate levels and ratios among the eight strains (Figures 4A,B). A time-course experiment analyzed different lengths of dark, anaerobic incubations and showed that a 3- or 4-day incubation period was long enough to produce sufficient quantities and ratios of succinate in 1299E (Figure 5). The succinate production rates from 1299E were 1.38 and 1.18 mg/L/h for 3- and 4-day incubation, respectively (Figure 5). Therefore, subsequent experiments were performed using a 3-day incubation period under dark, anaerobic conditions.

### A metabolome analysis

**Figure 6:**
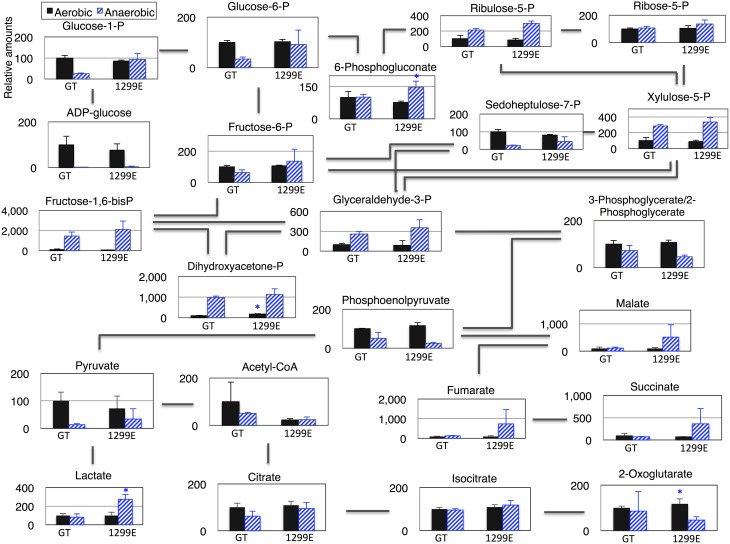
**Levels of metabolites in primary metabolism of the cyanobacterium ***Synechocystis*** 1299E strain, which overexpresses ***sigE*** and lacks ***ackA*****. Data represent means ± SD from three independent experiments. Metabolite levels were calibrated relative to that of corresponding metabolites in the wild-type strain (GT) under aerobic conditions (set at 100%). P designates phosphate. Asterisks indicate the statistically significant differences between GT and 1299E (Student's *t*-test; ^*^P < 0.05).

A metabolome analysis using the GT and 1299E strains grown under aerobic and anaerobic conditions was then performed to clarify the metabolic profiles. After dark, aerobic cultivation, ADP-glucoses disappeared and the levels of several sugar phosphates increased (Figure 6 and Table S1). The fructose-1, 6-bisphoshate and dihydroxyacetone phosphate levels increased more than 10 times under anaerobic conditions in the wild-type strain (Figure 6). Phosphoenolpyruvate, pyruvate and acetyl-CoA decreased greatly under anaerobic conditions (Figure 6). The levels of sugar phosphates, such as glucose-1-phosphate, glucose-6-phosphate, ribulose-5-phosphate, 6-phosphogluconate, fructose-6-phosphate, and fructose-1, 6-bisphosphate, in the 1299E strain under anaerobic conditions, were higher than those in the wild-type strain (Figure 6). Phosphoenolpyruvate and acetyl-CoA were lower in the 1299E strain than in the wild-type strain, and organic acids, such as succinate, lactate, malate, and fumarate, were higher in the 1299E strain than in the wild-type strain under anaerobic conditions (Figure 6).

The quantification of the glycogen levels before and after anaerobic cultivation for 3 days revealed that 33% of glycogen was consumed in the wild-type strain; however, 70% of glycogen were consumed in the 1299E strains (Table 1).

**Table 1:** **Relative glycogen levels in GT and 1299E**.

**Strain**	**Aerobic**	**Anaerobic**
GT	100 ± 13.1	67.6 ± 10.8
1299E	104.4 ± 1.7	29.5 ± 4.8

*Data represent means ± SD results from four independent experiments. Glycogen levels were calibrated relative to that in GT under light conditions (set at 100%). ND, glycogen under detectable levels*.

## Discussion

**Figure 7:**
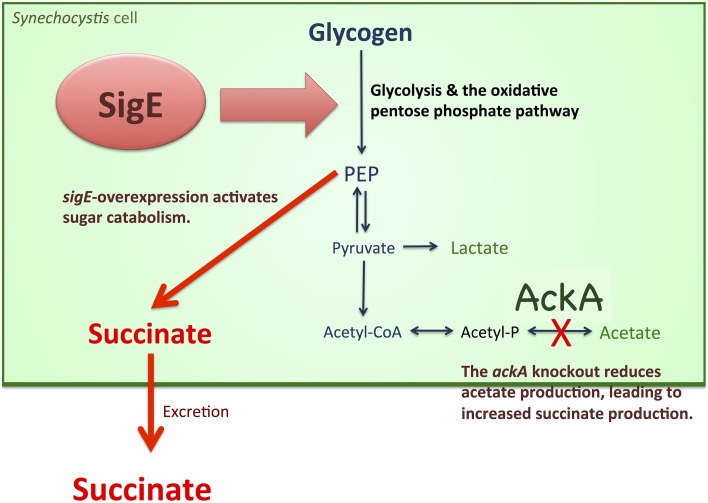
**Schematic model of succinate production from ***Synechocystis*** 6803**. *sigE* overexpression activates sugar catabolism, possibly leading to increased succinate production. The *ackA* knockout reduces acetate biosynthesis, resulted in increased production of succinate and lactate production.

Fermentation is closely related to sugar metabolism. In *Synechococcus* 7002, the levels of excreted fermentation products were altered by the disruption of *glgC*, which encodes ADP-glucose pyrophosphorylase (Guerra et al., 2013). Lactate production in the *glgC* knockout strain was approximately half of that in the wild-type, while acetate and alanine production were not significantly affected (Guerra et al., 2013). The reason for the decreased lactate excretion may be the slower catabolic rate of reduced sugars in the *glgC* knockout mutant (Guerra et al., 2013). The rate of sugar catabolism, not the amount of total reduced sugars, was important for the increased lactate production (Guerra et al., 2013). This finding was consistent with our results that *sigE* overexpression, which accelerated glycogen degradation and glucose catabolism (Osanai et al., 2011), increased succinate production in *Synechocystis* 6803 (Figures 4A, 7). Glycogen degradation was enhanced by *sigE* overexpression during dark, anaerobic conditions (Table 1), possibly leading to increases in the biosynthesis of intracellular organic acids (Figure 6 and Table S1), which in turn led to the production of extracellular succinate at a higher level (Figures 4A, 7).

A metabolome analysis revealed that the levels of fructose-1,6-bisphosphate and dihydroxyacetone phosphate increased more than 10 times after dark, anaerobic cultivation in the wild-type strain (Figure 6). Glyceraldehyde-3-phosphate production also increased, but 3-phosphoglycerate/2-phosphoglycerate, phosphoenolpyruvate, pyruvate, and acetyl-CoA decreased after dark, anaerobic cultivation in the wild-type strain (Figure 6). These results suggest that important enzymatic reactions exist downstream of glyceraldehyde-3-phosphate under dark, anaerobic conditions. Glyceraldehyde-3-phosphate dehydrogenase, which produces glycerate-1,3-bisphosphate from glyceraldehyde-3-phosphate, is encoded by *gap1* and *gap2*, and the reactions were uniquely catalyzed by the two enzymes in *Synechocystis* 6803. Gap1 catalyzes the catabolic reactions and Gap2 catalyzes the anabolic reactions (Koksharova et al., 1998). In addition, the *gap1* transcript levels are regulated by at least two transcriptional regulators, SigE and a response regulator Rre37 (Osanai et al., 2005; Azuma et al., 2011), indicating the importance of *gap1* in the sugar catabolism of *Synechocystis* 6803. The flux into the TCA cycle in *Synechocystis* is relatively low compared with other heterotrophic bacteria (You et al., 2015). Current results demonstrate that the flux into succinate can be up-regulated by our genetic modification under dark, anaerobic conditions.

A knockout of *ackA* reduced the acetate level but a knockout of *acs* did not affect the acetate level (Figure 4A), suggesting the major route of acetate biosynthesis under dark, anaerobic conditions is through an AckA-dependent pathway in this cyanobacterium (Figure 1). *Prochlorococcus* species lack *ackA* in their genomes (KEGG database URL: http://www.genome.jp/kegg-bin/show_pathway?syn00620), and thus, acetate biosynthesis pathway may be diverse among cyanobacteria. The mutant lacking *ddh* showed diminished lactate production, but lactate was produced by *sigE* overexpression even in the *ddh* knockout (Figure 4A). These results indicate another pathway for lactate biosynthesis exists in this cyanobacterium. Lactate can be synthesized from lactoylglutathione, which is derived from dihydroxyacetone phosphate (KEGG database URL: http://www.genome.jp/kegg-bin/show_pathway?syn00620), and the pathway may be activated by *sigE* overexpression. Our metabolome analysis revealed that NADPH disappeared, and that malate, fumarate, and succinate generally increased in the strains producing more succinate (Figure 6 and Table S1). These data suggest that succinate is produced through the reverse TCA cycle, as shown in Figure 1, as in other heterotrophic bacteria (Lee et al., 2006). Thus, the metabolic flux toward succinate production has been clarified in this cyanobacterium by the metabolome analysis. Pyruvate and phosphoenolpyruvate were severely reduced by light-to-dark transition (Iijima et al., 2015), and thus, the provision of these metabolites may be important to increase organic acid production under dark, anaerobic conditions. The current results demonstrated that a combination of the genetic manipulation of genes encoding a metabolic enzyme and a sigma factor succeeded in up-regulating the succinate levels. Future study about the detailed metabolic regulation will contribute to further understanding of the mechanistic implication of succinate excretion from this cyanobacterium.

### Conflict of interest statement

The authors declare that the research was conducted in the absence of any commercial or financial relationships that could be construed as a potential conflict of interest.
