# Supplementary material for: Genetic manipulation of a metabolic enzyme and a transcriptional regulator increasing succinate excretion from unicellular cyanobacterium
Source: Front Microbiol. 2015 Oct 6;6:1064. doi: 10.3389/fmicb.2015.01064 (PMC4594341; doi:10.3389/fmicb.2015.01064)
Supplement: Supplementary file 2 [file Table2.DOCX]

**Table S2** List of primers

| Gene name | | Forward primer | Reverse primer | | |
| --- | --- | --- | --- | --- | --- |
| Primers for amplification of pKRP10 and pKRP13 | | | | | |
| pKRP10 | 5′-AAATTTCTCGAGGAATTCGAGCTCGGTACC-3′ | | | | 5′-AAACCCGACGTCGACTCACTATAGGGAGAC-3′ |
| Primers for amplification of *ddh*, *acs*, and *ackA* regions for knock-in vectors | | | | | |
| *ddh* | 5′-TTCCGCATGCAACACAGGAGTTGGAGGC-3′ | | | 5′-TTAAGATATCAGCTCTTCCTCTTCTTCG-3′ | |
| *acs* | 5′-TTCCGCATGCGTGTACCGCTGATGTGG-3′ | | | 5′-TTAAGATATCTTAGCCCTCCCGTAATTT-3′ | |
| *ackA* | 5′-TTCCGCATGCAGAGATTGACCTGGTGG-3′ | | | 5′-TTAAGATATCGATTGCTTTCTCTGTCCC-3′ | |
| Primers for confirmation of knockout and *sigE* insertion | | | | | |
| pTCP1556 | | 5'-AAAGGGTAAGGGTGCAAAGG-3' | 5′-CGTTAATGACCAAGCTCC-3′ | | |
| pTCP0542 | | 5'-AAAGGGTAAGGGTGCAAAGG-3' | 5′-ATCACATCATCCACCCG-3′ | | |
| pTCP1299 | | 5'-AAAGGGTAAGGGTGCAAAGG-3' | 5′-TTCCATTGACCCGGCAA-3′ | | |
